# The Effect of Gelatine Packaging Film Containing a *Spirulina platensis* Protein Concentrate on Atlantic Mackerel Shelf Life

**DOI:** 10.3390/molecules25143209

**Published:** 2020-07-14

**Authors:** Nadia Stejskal, José M. Miranda, Josefa F. Martucci, Roxana A. Ruseckaite, Santiago P. Aubourg, Jorge Barros-Velázquez

**Affiliations:** 1 INTEMA, CONICET-UNMdP, Av. Colón 10850, 7600-Mar del Plata, Argentina; nadiastejskal@gmail.com (N.S.); jmartucci@fi.mdp.edu.ar (J.F.M.); roxana@fi.mdp.edu.ar (R.A.R.); 2 Department of Analytical Chemistry, Nutrition and Food Science, School of Veterinary Sciences, University of Santiago de Compostela, Av. Carballo Calero s/n, 27002-Lugo, Spain; josemanuel.miranda@usc.es (J.M.M.); jorge.barros@usc.es (J.B.-V.); 3 Department of Food Science and Technology, Marine Research Institute (CSIC), c/ Eduardo Cabello 6, 36208-Vigo, Spain

**Keywords:** *Spirulina platensis*, protein concentrate, gelatine film, packaging, refrigerated mackerel, microbial activity, lipid damage, quality

## Abstract

The use of packaging films containing natural preservative compounds attracts great attention for the quality improvement of seafood. Microalga spirulina (*Spirulina platensis*) represents a potential source of high added-value and preservative biocompounds. The goal of this study was to enhance the quality of refrigerated Atlantic mackerel (*Scomber scombrus*) by including a protein concentrate (PC) of spirulina in a gelatine-based film. Quality changes in fish muscle were monitored by microbial and chemical analyses throughout an 11-day refrigerated storage (4 °C). As a result of the presence of spirulina PC in the film, an antimicrobial effect (*p <* 0.05) was concluded as determined by comparative evolution of aerobes and psychrotrophs, while no effect (*p* > 0.05) was concluded for *Enterobacteriaceae*, proteolytics and lipolytics counts. Furthermore, a lower (*p* < 0.05) formation of trimethylamine and free fatty acids was detected. Lipid oxidation, measured by fluorescent compounds formation, also exhibited lower average values in fish corresponding to the batch containing spirulina concentrate. The preservative effects observed can be explained on the basis of the presence of antimicrobial and antioxidant compounds in the microalga concentrate. It is proposed that the current packaging system may constitute a novel and promising strategy to enhance the quality of commercial refrigerated fatty fish.

## 1. Introduction

Fish and fish products have long been recognized as a valuable source of high-quality digestible proteins, long-chain ω3 fatty acids, fat soluble vitamins (A and D), as well as essential minerals [1,2,3]. However, fish and fish products have shown to be especially prone to damage (i.e., endogenous enzyme activity, microbial decomposition and lipid oxidation) throughout the different steps involved in commercialization [4]. Thus, deterioration begins immediately upon catching or slaughtering and continues to varying degrees, depending especially on storage conditions. Among the different preserving strategies, fish refrigeration has represented a crucial strategy to provide high-quality fish for human consumption.

In order to increase the shelf life time of refrigerated fish, different strategies have been developed. One of the most recent has been the use of packaging films including preservative compounds (i.e., antimicrobials and antioxidants) so that a marine product with an increased shelf life time is attained [5,6]. Among preservative compounds, adverse health problems resulting of persistent consumption of synthetic ones have recommended the use of natural antioxidants and antimicrobials as an alternative [7,8,9]. Therefore, the identification and isolation of novel natural antioxidants from aquatic and terrestrial sources is currently receiving increasing attention.

One of such possibilities has been the use of macroalgae- and microalgae-derived products. Among them, spirulina (*Spirulina platensis*), a blue-green microalga, has recently attracted great attention as a massive cultured species in the world and for its extended use in aquaculture as a food supplement in fish diets [10]. Its biomass nutritional value represents a potential source of high added-value biocompounds such as carbohydrates (10–20% dry weight (d.w.), vitamins, pigments (3–7% d.w.), polyphenols, flavonoids, lipids (3–10% d.w.) and preservative protein-derived compounds (50–70%, d.w.) named phycobiliproteins [11,12].

On the basis of their wavelength absorption maxima, phycobiliproteins can be divided into four major classes, phycocyanin (*λ* 620 nm) being the most abundant, followed by phycoerythrin (*λ* 565 nm) and allophycocyanin (*λ* 650 nm) [13]. Phycobiliproteins are composed of a number of subunits, each one having a protein backbone and a phycobilin moiety linked by a covalent bond. Phycobilins are chromophores that are capable of capturing light and are the principal responsible for the photosynthetic activity [14]. It has been also reported that phycobilins may be one of the main components responsible for the bioactivity of phycobiliproteins [15]. As an example, phycocyanin extracted from *S. platensis* exhibited in vitro dose-dependent antioxidant activity as determined by bleaching, ferric ion reducing power and 2,2-diphenyl-1-picrylhydrazyl (DPPH) radical scavenging activity, as well as a great antibacterial action against foodborne pathogens such as *Staphylococcus aureus, Micrococcus luteus, Escherichia coli* and *Pseudomonas* spp [16]. Benehaldj et al. [17] characterized an *Arthrospira platensis* protein isolate able to form edible films using sorbitol as plasticizer. The same authors complexed this protein isolate with lysozyme to produce antimicrobial edible films where lysozyme release kinetics depended on the pH.

Related to marine products preservation, the employment of *S. platensis* extracts can be considered as scarce. Thus, only recent studies have reported the physicochemical characteristics of gelatine-derived films including a protein concentrate (PC) of spirulina [18,19]. Later on, the storage stability of a lean and highly appreciated fish species (hake, *Merluccius merluccius*) packaged in crosslinked-gelatine films containing a PC of *S. platensis* was analyzed [20]. Such active packaging system reduced microbial counts and delayed lipid damage of hake fillets stored at 4 °C. Results were explained on the basis of the preserving properties provided by the gelatine-based film including spirulina PC [18,19].

The present study is focused on a fatty and pelagic fish species (Atlantic mackerel, *Scomber scombrus*). However, it is recognized as a healthy food because it is a good source of high quality nutrients, particularly ω3 fatty acids [21], this species remains underutilized mainly because of its limited shelf life [22]. In this sense, previous reports have shown an important endogenous pro-oxidant activity [23] and a rapid quality loss during refrigerated storage [24]. Accordingly, in the present study, the effect of a gelatine-based film including a spirulina PC on the quality of refrigerated Atlantic mackerel muscle was evaluated. Quality changes were monitored by microbial and chemical analyses after 4 and 7 days of storage. To the best of our knowledge, the current research constitutes the first approach for the development of spirulina-based films for the quality enhancement of a fatty and underutilized fish species.

## 2. Results and Discussion

### 2.1. Comparative Analysis of Microbial Development in Fish Batches

According to the experimental design, three different fish batches were considered. In the first one, fish was packaged in a gelatine-based film including a spirulina PC (gelatine–spirulina treatment, SP batch). Furthermore, two different packaging systems were considered as control: a gelatine film without spirulina PC (control gelatine, GE batch) and a low-density polyethylene film (control polyethylene, CT batch).

**Figure 1:**
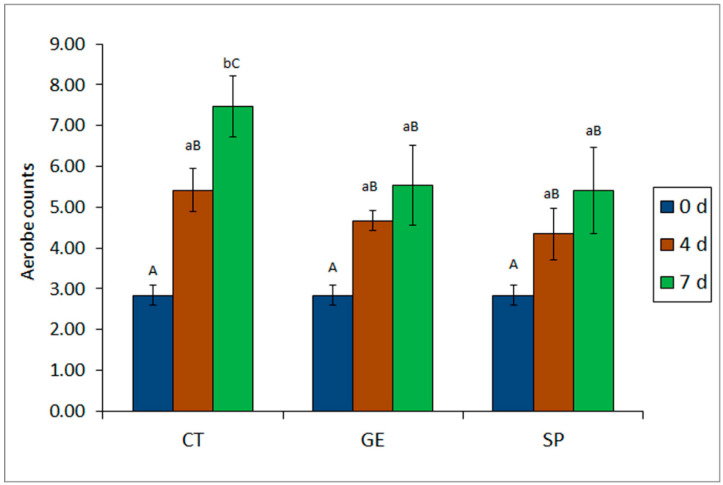
Aerobes count assessment (log CFU g^−1^ muscle) * in mackerel muscle stored under different packaging conditions **. * Average values of three replicates (*n* = 3); standard deviations are indicated by bars. Average values accompanied by different lower case letters (a, b) indicate significant differences (*p* < 0.05) as a result of packaging condition; average values accompanied by capital letters (A, B, C) indicate significant differences (*p* < 0.05) as a result of refrigeration time. ** Packaging conditions: CT (control polyethylene), GE (control gelatine) and SP (gelatine–spirulina).

All batches under study showed a progressive increase on aerobes counts throughout refrigerated storage (Figure 1). The investigation of aerobes in all three batches revealed a significant (*p* < 0.05) inhibitory effect derived of the presence of gelatine alone or combined with *S. platensis* PC in the packaging film, on the growth of this microbial group, when compared with the CT batch. Thus—and as can be observed in Figure 1—the SP batch exhibited the lowest microbial counts both at medium and advanced storage times. Remarkably, on day 7 the SP batch exhibited microbial numbers 2 log units below the CT control batch. However, the differences between SP and GE batches were not significant (*p* > 0.05), the former batch exhibited slightly lower values than the latter at both sampling times (Figure 1).

Psychrotrophs development also showed a marked increase with storage time in all batches, except for fish specimens corresponding to SP batch, in which a stabilizing effect of packaging between 4 and 7 days was observed (Table 1). The results indicated a significant (*p* < 0.05) inhibitory effect on psychrotrophs counts in SP batch derived from the presence of *S. platensis* PC in the packaging film at advanced storage time, as compared with GE and CT batches. As in the case of aerobes, both GE and SP batches exhibited lower microbial numbers than CT batch on day 7. The highest differences observed among batches reached a maximum of 3.77 log units, a result that indicated that the presence of spirulina PC in the packaging film reduced psychrotrophs growth by a factor above 5000 than the CT batch (Table 1).

With respect to *Enterobacteriaceae* growth, all batches exhibited values below 4 log CFU·g^−1^ at all sampling times (Table 1). As storage time progressed, slightly higher average *Enterobacteriaceae* counts were determined in fish specimens corresponding to GE and SP batches, while those belonging to the CT batch exhibited higher average increases. Interestingly, differences among batches were observed and resulted to be significant (*p* < 0.05) at advanced storage time (day 7). Thus, both gelatine-containing packaging films provided protection with respect to the activity of this microbial group as compared with the CT batch. Remarkably, both GE and SP batches exhibited counts below 2.0 log CFU·g^−1^ on day 7, while the CT batch rose up to nearly 3.50 log CFU·g^−1^ at this storage time (Table 1). These results indicated that gelatine-based films allowed the control of *Enterobacteriaceae* in mackerel muscle and that in this case the presence of spirulina PC in the packaging film did not provide any additional protective effect.

The investigation of specific spoilage microorganisms, namely proteolytic and lipolytic bacteria, was also performed and the results are compiled in Table 2. Proteolytic bacteria may negatively affect the stability of the myofibrillar fraction and, consequently, deteriorate fish texture [25], while lipolytic microorganisms have been reported to exhibit a marked effect on free fatty acids (FFA) formation, this leading to important deteriorative events such as texture modification and pro-oxidative mechanisms [4,26]. Table 2 shows a marked development of both microbial groups in all batches as storage time progressed. However, both GE and SP batches exhibited a better (*p* < 0.05) control of proteolytic bacteria than the CT batch at advanced storage time (day 7). The highest differences among batches (1.66 log units) were observed between SP and CT batches on day 7, this allowing to conclude a remarkable effect of the active gelatine packaging film containing spirulina PC on the growth of proteolytic bacteria.

With respect to lipolytic bacteria, a similar behavior was observed (Table 2). Thus, GE and SP batches exhibited significantly (*p* < 0.05) lower counts of this microbial group than the CT batch on day 7. It is remarkable that the highest differences were observed between SP and CT batches (1.72 log units), this indicating a notable inhibitory effect of the active gelatine film on the growth of bacteria able to hydrolyze lipid compounds. Consequently, the protective effect of gelatine-based packaging films was concluded, especially of the one including spirulina PC, on microbial-based lipolytic events in mackerel muscle.

Current results about microbial activity inhibition are in agreement with previous reports about the antimicrobial effects of *S. platensis* concentrates or extracts. Recently and closely related to the current study, the storage stability of hake (*M. merluccius*) fillets was enhanced by packaging in crosslinked-gelatine films containing *S. platensis* PC [20]; as a result, counts for aerobe mesophiles, psychrotrophs, proteolytics, lipolytics and *Enterobacteriaceae* were notably reduced. Similarly, although related to a macroalga (i.e., *Fucus spiralis*) concentrate, microbial counts (aerobes, psychrotrophs, *Enterobacteriaceae*, proteolytics, lipolytics and anaerobes) decreased in hake (*M. merluccius*) muscle stored at 4 °C when it was packaged with a gelatine-based film including a *Fucus spiralis* PC [27].

Concerning in vitro studies, phycocyanin extracted from *S. platensis* has been reported to exhibit a great in vitro antibacterial activity against foodborne pathogens such as *S. aureus, M. luteus, E. coli* and *Pseudomonas* spp [16]. Furthermore, based on an in vitro experiment, an antibacterial peptide (18 amino acid residues with a molecular mass of 1878.97 Da) isolated from an alkaline protease and papain hydrolysate of *S. platensis*, exhibited antimicrobial activity against *E. coli* and *S. aureus* [28]. Furthermore, hydrolysis of *S. platensis* proteins by trypsin and chymotrypsin enzymes revealed that approximately 20–22-kDa proteins and their derivative peptides were able to decrease in vitro bacterial growth (*E. coli* and *S. aureus*) [29].

### 2.2. Chemical Analyses of Quality Deterioration

A progressive increase of pH value was detected in all batches as storage time progressed (Table 3). Concerning the spirulina PC presence in the packaging film, no significant effect (*p* > 0.05) could be observed. Nevertheless, a lower average value was detected in mackerel muscle corresponding to the SP batch.

Trimethylamine–nitrogen (TMA–N) values (Table 3) determined in the initial fish (1.69 ± 0.14 mg·kg^−1^ muscle) can be considered as corresponding to high-quality specimens. A marked TMA–N increase (*p* < 0.05) was observed in all batches after 4 and 7 days of storage. At both sampling times, the presence of gelatine in the packaging film led to an inhibitory effect (*p* < 0.05) on TMA formation. Furthermore, fish specimens corresponding to the spirulina batch provided lower average TMA–N values that resulted to be significant (*p* < 0.05) at the end of the storage time.

Both pH and TMA are considered closely related indicators of microbial activity [4,20]. Thus, increases in the pH of the fish muscle indicate the accumulation of alkaline compounds, such as ammonia and other volatile amines such as TMA, all of them principally derived from microbial activity. Consequently, the values observed for pH and TMA–N in this study are in agreement with the microbial analyses presented above. Interestingly, it has been suggested that pH values above 7.0 may limit the shelf life of several fish species such as hake (*M. merluccius*) [30], although other species such as megrim (*Lepidorhombus whiffiagonis*) have shown acceptable quality above this pH value [31]. Nevertheless, none of the batches considered in this study surpassed the pH 7 value throughout storage time.

**Figure 2:**
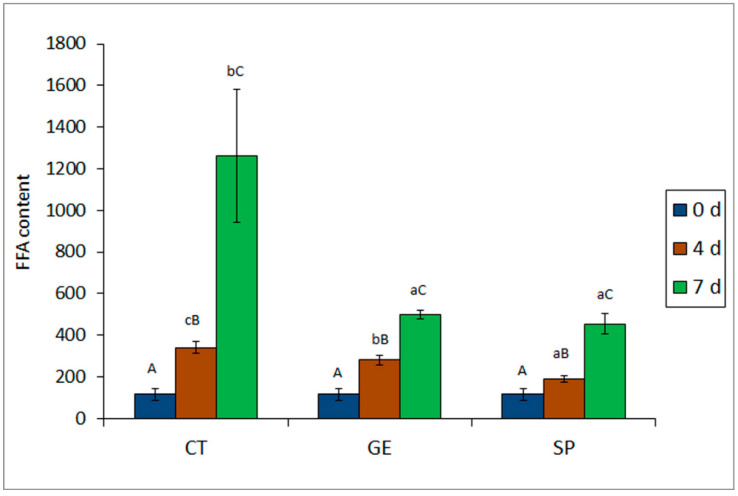
Evolution of free fatty acid (FFA) content (mg·kg^−1^ muscle) * in mackerel muscle stored under different packaging conditions **. * Average values of three replicates (*n* = 3); standard deviations are indicated by bars. Average values accompanied by different lower case letters (a, b, c) indicate significant differences (*p* < 0.05) as a result of packaging condition; average values accompanied by capital letters (A, B, C) indicate significant differences (*p* < 0.05) as a result of refrigeration time. ** Packaging conditions as expressed in Figure 1.

A marked FFA formation (*p* < 0.05) was observed throughout storage in all batches (Figure 2). After four days of storage, the lowest (*p* < 0.05) lipid hydrolysis rate was detected in fish corresponding to the SP batch. At the end of the storage time, the lowest average value was also detected in the spirulina batch, differences with respect to the control batch being significant (*p* < 0.05). In agreement to FFA formation, the growth of lipolytic bacteria was slowed down in fish corresponding to the SP batch, such differences being significant when compared to the CT batch (Table 2).

FFA formation in fish muscle during refrigerated storage has been explained as a result of both endogenous and microbial enzyme activities [26,32]. Before the end of the microbial lag phase, FFA formation is mostly caused by endogenous enzyme activity (i.e., lipases and phospholipases); later on, microbial activity is the predominant mechanism of FFA generation. On the basis that a strong development of FFA formation has been observed in the present study at both sampling times, microbial activity seems to be the most relevant mechanism responsible of FFA formation.

Previous studies reporting the effects of spirulina extracts, either proteinaceous or not, on lipid hydrolysis development in seafood are scarce. Thus, an inhibitory effect on FFA formation was observed in hake (*M. merluccius*) muscle packaged in a crosslinked-gelatine film including a spirulina PC [20]. Likewise, the presence of a PC from a macroalga species (*F. spiralis*) in a gelatine-based packaging system implied a lower development of lipid hydrolysis in hake (*M. merluccius*) muscle [27].

Determination of fluorescent compounds showed a progressive increase with storage time in all batches (Table 3). Lower average values were determined in fish corresponding to both gelatine-containing batches, such values being lower in the case of fish corresponding to the spirulina PC batch. However, no significant differences (*p* > 0.05) among batches could be outlined related to these lipid oxidation events. As an explanation for this slight effect, it could be mentioned that lipid oxidation should not be an especially relevant damage pathway in a refrigerated storage experiment such as the present one. Furthermore, the fact that mackerel specimens exhibited a relatively low lipid content (16.5 ± 6.2 g·kg^−1^ muscle), according to the fact of being caught in Spring [33], supports this statement.

A previous study has pointed out the antioxidant effect of *S. platensis* extracts or concentrates on different kinds of seafood on the basis of the presence of a wide range of antioxidant molecules (i.e., phycocyanins, polyphenols, flavonoids) [34,35]. Thus, the presence of a spirulina PC in a gelatine-based film led to a higher retention of polyunsaturated fatty acids in hake (*M. merluccius*) muscle during refrigerated storage [20]. Additionally, phycocyanin obtained from *S. platensis* exhibited antioxidant properties in dried, salted fish (Pacu, *Piaractus mesopotamicus*) during a 60-day storage period at 25 °C [36]. Furthermore, phycocyanin extracted from *S. platensis* exhibited in vitro dose-dependent antioxidant activity as determined by bleaching, ferric ion reducing power and 2,2-diphenyl-1-picrylhydrazyl (DPPH) radical scavenging activity [16]. Related to macroalgae, the inclusion of *F. spiralis* PC in a gelatine-based film inhibited lipid oxidation mechanisms in refrigerated fish [27].

## 3. Materials and Methods

### 3.1. Comparative Analysis

Commercially available *Spirulina platensis* (lyophilized powder, Martin Bauer GmbH & Co, Vestenbergsgreuth, Germany) was used as raw material without further treatment. Bovine gelatine type B, isoionic point, pI 5.3, Bloom 150, was kindly provided by Rousselot Argentina (Villa Tesei, Argentina). Glycerol analytical grade (Gly, 98%) was purchased from Anedra (Buenos Aires, Argentina) and used as a plasticizer. Sodium alginate with moisture content ≤ 15.0% and pH = 6.5–8.6 was purchased from Sigma-Aldrich (St. Louis, MO, USA). Phosphate buffer (pH 10), sodium hydroxide and hydrochloric acid were obtained from Anedra (Buenos Aires, Argentina).

All other solvents and chemical reagents used in the current study were of reagent grade (Merck, Darmstadt, Germany).

### 3.2. Preparation and Physicochemical Characteristics of Film Systems

*S. platensis* was subjected to an extraction protocol, based on repeated aqueous, alkaline and acidic extraction steps followed by several rounds of centrifugation and recovery using precipitation and ultracentrifugation [17,37]. For it, 5 g of commercially lyophilized powder of *S. platensis* were dissolved in 100 mL of phosphate buffer pH 10 (0.005 mol·L^−1^) under agitation (500 rpm, 60 min). Subsequently, the suspension was centrifuged (10,000× *g*, 10 min, 3 times) using an ultracentrifuge (Sartorius 4–15). The supernatant was collected (batch A) and the pellet was dissolved and centrifuged again under the same conditions as described above, to obtain batch B. Both supernatants (batches A and B) were pooled together and the pH decreased from 10.0 to 3.0 by the addition of 0.1 mol·L^−1^ aqueous HCl solution to precipitate the protein fraction. The suspension was centrifuged (10,000× *g*, 30 min) and the precipitated proteins were freeze-dried (VirTis Bench Top SLC lyophilizer, Warminster, PA, USA), this leading to the *S. platensis* PC.

Films were produced by casting from their film-forming solutions (FFS) [38], using glycerol as plasticizer and oxidized sodium alginate (OA) as efficient crosslinking agent according to Stejskal et al. [20]. Active films were produced by dissolving 8 g of gelatine and 2 g of the freeze-dried PC in 100 mL of phosphate buffer solution pH 10 under stirring at 40 °C. Glycerol (30% wt. on dry protein basis) and OA (5% wt. on dry protein basis) were incorporated to the FFS, the suspension being stirred at 40 °C for 120 min. Then, the FFS were cast onto Teflon-coated Petri dishes (diameter 10 cm) and dried at 40 °C in a convection oven at controlled relative humidity, until constant weight. Films were conditioned for 48 h in a chamber at 4 ± 1 °C prior to analysis. The resulting film was named as gelatine–spirulina condition (SP batch).

Two different control-packaging batches were taken into account in the current study. First, a control batch was prepared as mentioned above, but consisting of the gelatine film without spirulina PC (control gelatine, GE batch). Second, a low-density polyethylene film was also employed as control (control polyethylene, CT batch). For both control systems, 10-cm-packaging films were prepared as for the SP batch. The low-density polyethylene used to elaborate the packaging films (CT batch) had a water vapor transmission rate of 3.62 g·m^−2^·d^−1^ when measured at 38 °C and 90% relative humidity and a thickness of 140 μm [39].

Previous reports described the characteristics and properties of spirulina PC-derived films [18,19]. Thus, the incorporation of 2% PC (dry-protein basis) provided antimicrobial activity against *E. coli* and *S. aureus* (inhibition halo for both pathogens 10 ± 1 mm) and antioxidant activity (radical scavenging activity, RSA: 121.5 ± 5.7 vs. 21.2 ± 6.8 µg ascorbic acid·g^-1^ for the active and control gelatine film, respectively) [18]. In addition, the UV-visible barrier properties were enhanced by the presence of PC in the films. The resulting SP film was thicker (373 ± 42 vs. 320 ± 40 μm), 35% more stretchable (195 ± 8 vs. 145 ± 27%), 78% more mechanically resistant (2.5 ± 0.1 vs. 1.4 ± 0.2 MPa) and less permeable to water vapor (WVP = 7.2 ± 0.3 10^−15^ kg·m·h^−1^·Pa^−1^ and WVP = 8.8 ± 0.3 10^−15^ kg·m·h^−1^·Pa^−1^) than control gelatine counterpart films (GE batch).

The content of spirulina PC employed in the present study was based on several preliminary tests carried out in our laboratory. Thus, concentrations higher than 20 g spirulina PC·L^−1^ buffer led to negative modifications in fish sensory descriptors such as muscle color and odor and to non-suitable mechanical properties of the films (data not shown).

### 3.3. Fish Material, Processing and Sampling

Fresh Atlantic mackerel (*Scomber scombrus*) specimens (45) were caught in May 2018 near the Galician Atlantic coast (northwestern Spain) and transported to the laboratory. Throughout this process (10 h), the fish specimens were maintained in ice. The length and weight of the fish specimens ranged from 27 to 32 cm and from 215 to 265 g, respectively.

Upon arrival to the laboratory, nine individual fish specimens were separated and analyzed as initial material (day 0). These fish specimens were divided into three different groups (three specimens per group), the white muscle from the fish back location being analyzed independently in each group (three replicates; *n* = 3). The remaining 36 fish specimens were distributed into three batches (12 specimens per batch). In each batch, fish specimens were filleted and cut into pieces (ca. 35 g). Three fish pieces were packaged from each specimen, all pieces being taken from the back muscle. Then, fish pieces corresponding to all three batches were seal-packaged individually in the three above-mentioned packaging systems (CT, GE and SP batches; 36 fish pieces for each packaging condition), respectively and placed inside a refrigerated room (4 °C). Packaged fish pieces from all batches were stored for a 7-day period, sampling and analyses being carried out on days 4 and 7. At each sampling time, 18 packaged fish pieces were taken from each batch for analysis and divided into three groups (six packaged fish pieces in each group), the white muscle being studied independently in each group (three replicates; *n* = 3).

### 3.4. Microbial Analyses

Samples of 10 g were aseptically taken from fish muscle and mixed with 90 mL of 0.1% peptone water (Merck, Darmstadt, Germany). The mixture was homogenized in sterilized stomacher bags (ES, Combourg, France) as described elsewhere [40,41]. The extracts were diluted in 0.1% peptone water.

The investigation of aerobes was carried out on plate count agar (PCA) (Oxoid Ltd., London, UK) once the plates was incubated at 30 °C for 48 h. The investigation of psychrotrophs was also carried out in PCA medium, but incubation was extended to 7 days and temperature reduced to 7–8 °C. *Enterobacteriaceae* growth was determined in Violet Red Bile Agar (VRBA) (Merck, Darmstadt, Germany) once the plates were incubated at 37 ± 0.5 °C for 24 h. Presence of specific spoilage microorganisms exhibiting proteolytic or lipolytic phenotypes was determined in casein-agar or tributyrin-agar, respectively, once the plates was incubated at 30 °C for 48 h, as described elsewhere [42].

Microbial analyses were carried out in triplicate. In all cases, microbial counts obtained were transformed into log colony-forming units (CFU) g^−1^ muscle values before undergoing the statistical analysis.

### 3.5. Chemical Analyses of Quality Deterioration

Different chemical analyses were carried out in order to assess microbial activity (pH and TMA determinations) as well as both lipid hydrolysis (FFA content) and oxidation (fluorescent compounds formation).

The evolution of pH values in mackerel muscle over storage time was determined using a 6-mm-diameter insertion electrode (Crison, Barcelona, Spain).

Trimethylamine-N values were determined using the picrate spectrophotometric (410 nm) method, as previously described by Tozawa et al. [43]. This method involved the preparation of a 5% trichloroacetic acid extract of fish white muscle (10 g in 25 mL). Results were expressed as mg TMA–N·kg^−1^ muscle.

Lipids were extracted from mackerel white muscle by the Bligh and Dyer [44] method, which employs a single-phase solubilization of the lipids using a chloroform–methanol (1:1) mixture. Results were expressed as g lipid·kg^−1^ muscle.

Free fatty acids content was determined using the lipid extract of the fish muscle by the Lowry and Tinsley [45] method, which is based on complex formation with cupric acetate-pyridine followed by spectrophotometric (715 nm) assessment. Results were expressed as g FFA·kg^−1^ lipids.

The formation of fluorescent compounds (Fluorimeter LS 45; PerkinElmer España; Tres Cantos, Madrid, Spain) was determined in the lipid extract of the fish muscle by measurements at 393/463 and 327/415 nm as described previously [33]. The relative fluorescence (RF) was calculated as follows: RF = *F/F_st_*, where *F* is the fluorescence measured at each excitation/emission wavelength pair and *F_st_* is the fluorescence intensity of a quinine sulfate solution (1 µg·mL^−1^ in 0.05-M H_2_SO_4_) at the corresponding wavelength pair. Results are given as the fluorescence ratio (FR), which was calculated as the ratio between the two RF values: FR = RF_393/463 nm_/RF_327/415 nm_.

### 3.6. Statistical Analysis

Data obtained from all microbiologic and chemical analyses were subjected to the ANOVA method to explore differences resulting from the effect of the packaging system and the refrigeration time. The comparison of means was performed using the least-squares difference (LSD) method. In all cases, analyses were carried out using the PASW Statistics 18 software for Windows (SPSS, Inc., Chicago, IL, USA); differences among packaging batches were considered significant for a confidence interval at the 95% level (*p* < 0.05).

## 4. Conclusions

The goal of this study was to enhance the quality of refrigerated Atlantic mackerel by the novel application of a PC from microalga spirulina in a gelatine-based packaging film. A remarkable antimicrobial effect of such film on aerobes and psychrotrophs activity was concluded. Moreover, a lower formation of TMA and FFA as well as the slowdown of lipid oxidation mechanisms, as determined by fluorescent compounds formation, in fish packaged in SP films, were also concluded. As a matter of fact, the preservative effects observed can be explained on the basis of the presence of antimicrobial and antioxidant compounds from the spirulina PC in the active packaging film.

The results of the present study underline the potential application of microalga *S. platensis* as a source of bioactive compounds for the seafood industry. Consequently, the proposed active packaging system can constitute a novel and promising strategy to enhance the quality of commercial refrigerated fatty fish and opens the way to its practical application. Further research related to the optimization of processing conditions (i.e., concentration of spirulina PC, film matrix and marine species concerned) will surely contribute to attain the best quality in other fatty fish products.

## Figures and Tables

**Table 1:** Psychrotrophs and *Enterobacteriaceae* count assessment (log CFU·g^−1^ muscle) * in mackerel muscle stored under different packaging conditions **.

Microbial Index	Refrigeration Time (days)	Packaging Condition		
		**CT**	**GE**	**SP**
**Psychrotrophs**	0	2.20 A (0.17)	2.20 A (0.17)	2.20 A (0.17)
	4	5.27 aB (1.01)	4.28 aB (0.72)	4.59 aB (0.43)
	7	8.48 cC (0.16)	6.59 bC (0.45)	4.71 aB (0.60)
**Enterobacteriaceae**	0	1.00 A (0.05)	1.00 A (0.05)	1.00 A (0.05)
	4	2.60 aAB (1.47)	1.69 aAB (0.65)	1.79 aAB (0.75)
	7	3.46 bB (0.15)	1.79 aB (0.28)	1.93 aB (0.81)

* Average values of three replicates (*n* = 3); standard deviations are indicated in brackets. For each microbial parameter, average values followed by different lower-case letters (a, b, c) indicate significant differences (*p* < 0.05) as a result of packaging condition; average values followed by capital letters (A, B, C) indicate significant differences (*p* < 0.05) as a result of refrigeration time. ** Packaging conditions: CT (control polyethylene), GE (control gelatine) and SP (gelatine–spirulina).

**Table 2:** Proteolytics and lipolytics count assessment (log CFU·g^−1^ muscle) * in mackerel muscle stored under different packaging conditions **.

Microbial Index	Refrigeration Time (days)	Packaging Condition		
		**CT**	**GE**	**SP**
**Proteolytics**	0	2.16 A (0.28)	2.16 A (0.28)	2.16 A (0.28)
	4	5.11 bB (0.18)	4.11 aB (0.35)	4.78 abB (0.94)
	7	7.29 bC (0.12)	5.71 aC (0.47)	5.63 aB (0.59)
**Lipolytics**	0	2.00 A (0.00)	2.00 A (0.00)	2.00 A (0.00)
	4	3.23 aB (0.83)	2.28 aA (0.49)	3.13 aB (0.76)
	7	5.85 bC (0.13)	4.26 aB (0.24)	4.13 aB (0.49)

* Average values of three replicates (*n* = 3); standard deviations are indicated in brackets. For each microbial parameter, average values followed by different lower case letters (a, b) indicate significant differences (*p* < 0.05) as a result of packaging condition; average values followed by capital letters (A, B, C) indicate significant differences (*p* < 0.05) as a result of refrigeration time. ** Packaging conditions as expressed in Table 1.

**Table 3:** Determination of different chemical quality indices * in mackerel muscle stored under different packaging conditions **.

Chemical Index	Refrigeration Time (days)	Packaging Condition		
		**CT**	**GE**	**SP**
**pH**	0	6.53 A (0.10)	6.53 A (0.10)	6.53 A (0.10)
	4	6.68 aAB (0.10)	6.63 aA (0.08)	6.67 aA (0.07)
	7	6.77 aB (0.08)	6.74 aA (0.15)	6.69 aA (0.08)
**Trimethylamine (TMA) (mg TMA–N kg^−1^ muscle)**	0	1.69 A (0.14)	1.69 A (0.14)	1.69 A (0.14)
	4	33.29 bB (18.41)	15.64 aB (3.02)	13.61 aB (2.61)
	7	619.69 cC (162.83)	98.55 bC (16.05)	72.40 aC (4.53)
**FR value**	0	1.42 A (0.15)	1.42 A (0.15)	1.42 A (0.15)
	4	2.75 aB (0.23)	2.46 aB (0.19)	2.06 aAB (0.53)
	7	3.22 aB (0.27)	2.96 aC (0.14)	2.83 aB (0.17)

* Average values of three replicates (*n* = 3); standard deviations are indicated in brackets. For each chemical parameter, average values followed by different lower case letters (a, b, c) indicate significant differences (*p* < 0.05) as a result of packaging condition; average values followed by capital letters (A, B, C) indicate significant differences (*p* < 0.05) as a result of refrigeration time. ** Packaging conditions as expressed in Table 1.
